# Unique Tropism and Entry Mechanism of Mumps Virus

**DOI:** 10.3390/v13091746

**Published:** 2021-09-01

**Authors:** Marie Kubota, Takao Hashiguchi

**Affiliations:** 1 Department of Otorhinolaryngology, Graduate School of Medical Sciences, Kyushu University, Fukuoka 812-8582, Japan; 2 Laboratory of Medical Virology, Institute for Frontier Life and Medical Sciences, Kyoto University, Kyoto 606-8507, Japan

**Keywords:** mumps virus, tropism, entry, glycan receptors, attachment protein, structure

## Abstract

Mumps virus (MuV) is an important human pathogen that causes parotitis, orchitis, oophoritis, meningitis, encephalitis, and sensorineural hearing loss. Although mumps is a vaccine-preventable disease, sporadic outbreaks have occurred worldwide, even in highly vaccinated populations. MuV not only causes systemic infection but also has a unique tropism to glandular tissues and the central nervous system. In general, tropism can be defined by multiple factors in the viral life cycle, including its entry, interaction with host factors, and host-cell immune responses. Although the underlying mechanisms of MuV tropism remain to be fully understood, recent studies on virus–host interactions have provided insights into viral pathogenesis. This review was aimed at summarizing the entry process of MuV by focusing on the glycan receptors, particularly the recently identified receptors with a trisaccharide core motif, and their interactions with the viral attachment proteins. Here, we describe the receptor structures, their distribution in the human body, and the recently identified host factors for MuV and analyze their relationship with MuV tropism.

## 1. Introduction

Mumps is a well-known infectious disease caused by the mumps virus (MuV). It occurs worldwide, with an average of 500,000 cases reported annually. After an incubation period of 2–4 weeks, mild symptoms of the infection appear, including low fever, headache, and malaise, typically followed by bilateral swelling of the parotid glands. Following the initial viral infection in the upper respiratory mucosa and regional lymph nodes, MuV spreads systemically throughout the body [1,2,3]. MuV shows tropism to glandular and neural tissues, to organs, such as the kidney and heart, and to joint tissues. Parotitis occurs in 60–70% of infections. Infrequently, other salivary glands, such as the submandibular and sublingual glands, may also be affected. Orchitis is found in 20–30% of infected adult males, mostly in conjunction with epididymitis. Approximately 15% of orchitis cases may progress to testicular atrophy, leading to decreased fertility [2,4]. Oophoritis is observed in 5% of infected adult females. Pancreatitis has been reported in 4% of cases. Central nervous system (CNS) involvement is another common manifestation of MuV infection. The most common neurological symptom is aseptic meningitis, which occurs in 1–10% of cases. Cerebrospinal fluid (CSF) pleocytosis has been reported in up to 50% of infected cases, with increased cytokine levels reflecting meningeal inflammation [2,5,6]. Encephalitis is another manifestation that is observed less commonly. Sensorineural hearing loss is a well-known complication, previously estimated to occur in 1/15,000 to 1/20,000 cases; however, more recent studies have suggested that the frequency could be 1/1000 [3,7]. In the majority of cases, mumps-related hearing loss is profound and resistant to therapeutics [8]. Live attenuated mumps vaccines, for example, the measles–mumps–rubella (MMR) vaccines, can prevent MuV infections in childhood and serious complications in young individuals [3].

Despite its unique tropism to glandular and neural tissues, the mechanisms underlying tropism are still poorly understood. Tropism is affected at multiple stages of the viral life cycle in response to interactions with host cells. Binding to cellular receptors during the initial stages of viral entry is known to be one of the determinants that plays a key role in regulating tropism and virulence of the virus.

**Figure 1:**
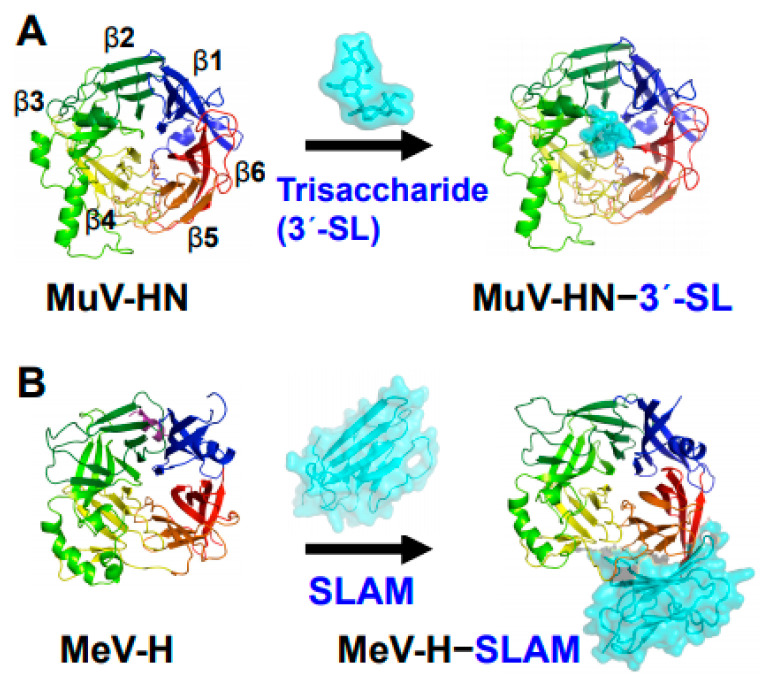
Structures of MuV and MeV attachment proteins bound to their receptors. (**A**) MuV-HN (rainbow color) bound to a glycan receptor analog (cyan) α2,3-linked sialyllactose (3′-SL) at the top receptor-binding pocket of the head domain; (**B**) MeV-H (rainbow color) bound to a proteinaceous receptor SLAM (cyan) at the lateral side of the head domain. The head domains of both MuV-HN and MeV-H monomers exhibit a six-bladed β-propeller fold.

MuV is an enveloped, non-segmented, negative-stranded RNA virus of the genus *Orthorubulavirus* and family *Paramyxoviridae*. Its genome contains 15,384 nucleotides encoding nucleocapsid (N), phosphor (P), matrix (M), small hydrophobic (SH), large (L), hemagglutinin-neuraminidase (HN), and fusion (F) proteins [3]. Based on the sequence of SH and HN genes, MuV has been classified into 12 genotypes [9]. The N protein together with the RNA-dependent RNA polymerase complex comprising L and P proteins form the ribonucleoprotein (RNP) complex, which plays an essential role in viral replication and transcription [1,10]. The M protein is involved in organizing virion assembly and supporting the budding of viral particles [1,10]. In some paramyxoviruses, M proteins have been proposed to inhibit host factors to enhance viral production [10,11]. SH proteins are present in *Rubulavirinae* such as MuV, parainfluenza virus 5 (PIV5), and Jeilongvirus [10]. SH proteins are believed to facilitate evasion of the host antiviral response by blocking the TNF-α-mediated apoptosis pathway [1,3]. Cellular entry of paramyxoviruses relies on two envelope glycoproteins: attachment proteins (HN in the majority of paramyxoviruses, including MuV (Figure 1A); hemagglutinin (H) in *Morbillivirus* (e.g., measles virus (MeV)) (Figure 1B), and glycoprotein (G) in *Henipavirus* (e.g., Nipah virus)), and F protein. While the attachment proteins (HN/H/G proteins) are responsible for receptor recognition, the F protein plays a role in membrane fusion [10].

Here, we review the recent advances in the general understanding of MuV entry based on the structures of MuV-HN and glycan receptor complexes, in combination with the functional findings of these interactions. We present the glycan receptors of MuV, their structural variations, and their interactions with the HN protein. In addition, we discuss MuV tropism based on the distribution of glycan receptors in the human body and the functional roles of recently identified host factors for the tropism of MuV.

## 2. Glycans and Viruses

Glycans on the cell surface are common receptors for a wide range of viruses, including influenza viruses, adeno-associated viruses, and rotaviruses. MuV also utilizes glycans for cellular entry, similar to other paramyxoviruses, such as human parainfluenza viruses (hPIVs), Newcastle disease virus (NDV), and Sendai virus [10]. Glycans exist mostly in the form of glycoproteins, glycolipids, or proteoglycans throughout the animal body and encompass a large number of molecules due to the combination of various monosaccharide components and their modifications, linkage types, and frameworks. Glycoproteins carry glycans covalently attached to the polypeptide backbone via *N*- or *O*-linkages [12]. *N*-glycans are attached to proteins by an *N*-glycosidic bond at the asparagine residue in the common amino acid consensus sequence of Asn-X-Ser/Thr and are composed of the core glycan structure of Manα1-3(Manα1-6)Manβ1-4GlcNAcβ1-4GlcNAcβ1. They form complex structures via elongation, branching, and modification. *O*-glycans are initiated at GalNAc and attach to the Ser or Thr residues of glycoproteins. Mucin is produced in epithelial tissues and is a major component of mucus in various tissues, such as the respiratory tract, salivary glands, gastrointestinal tract, reproductive organs, and urinary tract. It is known to contain a large number of *O*-glycans [13]. Glycosphingolipids (GSLs) are the major glycolipids in animals and are typically attached to ceramide (Cer) with β-linked galactose (GalCer) or glucose (GlcCer) [12]. Termini of glycan branches are often capped with α-linked sialic acid; the sialylated terminal structures are common to *N*-glycans, *O*-glycans, and glycolipids [12] and serve as common receptors for some viruses [14,15,16,17,18,19,20,21,22,23,24,25,26,27,28,29,30,31,32,33,34,35,36,37,38,39]. Sialic acids are typically linked to the C-3 or C-6 position of galactose via a C-2 carbon (called α2,3-linked or α2,6-linked sialic acid) or to the C-8 position of another sialic acid [12]. Some viruses, including influenza viruses, enteroviruses, and paramyxoviruses, are known to selectively bind to a specific linkage type of sialic acid [28,29,30,31,32,34,35,36,37,38]. For example, the attachment protein (hemagglutinin, HA) of human influenza viruses preferentially recognizes α2,6-linked sialic acid, whereas that of avian influenza viruses recognizes α2,3-linked sialic acid. The human upper respiratory tract contains a relatively high density of α2,6-linked sialic acids, whereas the avian intestinal tract contains a high concentration of α2,3-linked sialic acids. Therefore, selectivity of sialic acids has been hypothesized to possibly define the host range of the virus in humans and avians [40,41]. Some studies have also highlighted the correlation between glycan structure, which includes sugars beyond the terminal sialic acids, and viral receptor selectivity, providing an additional structural perspective regarding HA–glycan interactions [42,43,44,45].

## 3. Core Trisaccharide Receptor of MuV and Its Interaction with the MuV-HN Protein

MuV-HN is a type II membrane glycoprotein comprised of an N-terminal cytoplasmic tail, a transmembrane region, a stalk, and a C-terminal receptor-binding head domain. The head domain of the MuV-HN monomer exhibits a six-bladed β-propeller fold, which shows a specific affinity for glycans containing α2,3-linked sialic acid (Figure 1A). The selective binding of MuV-HN to α2,3-linked sialic acid was confirmed by using small-scale “hand-made” glycan arrays with glycoconjugates representing major sialylated glycans found in the human respiratory tract [28,46,47] and by binding experiments using a glycan array from the Consortium for Functional Glycomics (CFG) (http://www.functionalglycomics.org, 2 August 2021) [36,48]. In addition, the X-ray crystal structure of the MuV-HN head domain with a glycan receptor analog α2,3-linked sialyl lactose (3′-SL) proved that the top pocket of the β-propeller structure recognizes the glycan receptor. Although 3′-SL or α2,6-linked sialyl lactose (6′-SL) was co-crystallized with the MuV-HN protein, no electron density of 6′-SL was observed, indicating that MuV-HN preferentially binds to glycans containing α2,3-linked sialic acids [36].

**Figure 2:**
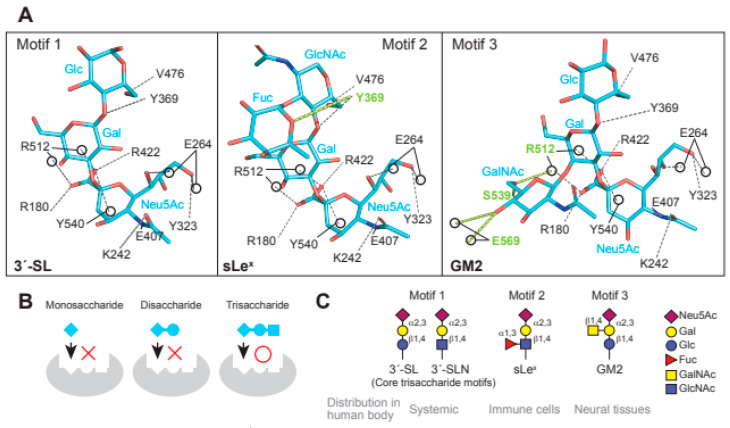
Glycan receptors with trisaccharide cores for MuV. (**A**) Interaction of the three receptor glycan motifs, 3′-SL (motif 1), sLe^x^ (motif 2), and GM2 (motif 3), with the amino acid residues of MuV-HN. The interactions between MuV-HN and the core trisaccharide moieties of 3′-SL (Neu5Acα2,3Galβ1,4Glc), sLe^x^ (Neu5Acα2,3Galβ1,4GlcNAc), and GM2 (Neu5Acα2,3Galβ1,4Glc) are completely conserved; (**B**) The sialyl-trisaccharide exhibits a higher affinity for MuV-HN compared to that of the sialyl-disaccharide. The trisaccharide structure is a necessary core-receptor structure for MuV; (**C**) Schematics of the three receptor motifs and their distributions in the human body.

In the interaction between MuV-HN and glycan receptors, the non-reducing terminal sialic acid (Sia-1) was shown to interact with five of the seven “active site residues”. The residues are highly conserved in viral and cellular sialidases and are involved in sialic acid interactions [49]. In addition to the active site residues, namely Arg180, Glu407, Arg422, Arg512, and Tyr540, Sia-1 in 3′-SL was shown to interact with the highly conserved amino acid residues Lys242, Glu264, and Tyr323 (Figure 2A). Most importantly, the direct contact with MuV-HN was mediated not only by Sia-1, but also by galactose (Gal-2) and glucose (Glc-3) moieties in the second or third position from the non-reducing terminus of glycans (Figure 2A). These observations demonstrated that the trisaccharide structure is responsible for the interaction between glycan receptors and the MuV-HN protein. The MuV-HN−3′-SL interaction is stabilized by an interaction between Glc-3 of 3′-SL and Tyr369 of MuV-HN (Figure 2A, motif 1). The stacking interaction of Tyr369 with the neighboring Phe370 and Tyr268 stabilizes the interaction with Glc-3. Importance of this interaction was supported by the observation that the Tyr369Ala substitution significantly reduced the fusion-supporting activity in the fusion assay performed to assess the function of MuV-HN and MuV-F proteins. The binding affinities between MuV-HN and glycan receptors, quantified by thermodynamic analysis using isothermal titration calorimetry (ITC), showed the equilibrium dissociation constant (Kd) of MuV-HN and disaccharide (Siaα2,3Gal) to be approximately 500 μM. In contrast, the Kd of MuV-HN and trisaccharide (Siaα2,3Galβ1,4Glc) was approximately 50 μM, indicating a 10-fold stronger interaction of MuV-HN with the trisaccharide compared to that with the disaccharide, hence suggesting the importance of the trisaccharide structure (Figure 2B). In a recent study, the interactions between MuV-HN and glycan receptors were characterized using ligand-based nuclear magnetic resonance (NMR) techniques [50]. Saturation transfer difference (STD) NMR analysis, which has the advantage of identifying and mapping the structure and affinity of a small-molecule ligand in receptor–ligand interactions [51,52], confirmed that MuV-HN interacts with the second and third moieties of a trisaccharide (NeuAcα2,3Galβ1,4GlcNAc: Sia1-Gal-2-GlcNAc-3) in addition to Sia-1 [50], in accordance with the findings from the X-ray crystal structure. In Sia-1, the *N*-acetyl group and proton H-8 were found to be strongly involved in the interaction with MuV-HN. Gal-2 interacts with MuV-HN through Val476 and Tyr369 residues and GlcNAc-3 interacts with Tyr369 and His205 residues of MuV-HN, although the interactions of Gal-2 and GlcNAc-3 with MuV-HN were weaker than those of Sia-1 with MuV-HN. Structural analysis using a longer sialylglycan (NeuAcα2,3Galβ1,4GlcNAcβ1,2Manα1,6(NeuAcα2,3Galβ1,4GlcNAcβ1,2Manα1,3)Manβ1, 4GlcNAcβ1,4GlcNAcβ-Asn) showed the epitope interacting with the MuV-HN protein to be mapped only to the trisaccharide portion at the non-reducing terminus [50]. This analysis confirmed that the trisaccharide structure is the core-receptor unit and that elongation of the glycan does not affect its binding with MuV-HN. Glycan array analyses and NMR studies have shown that branching of glycans reduces their affinity to MuV-HN [36,50], presumably due to steric hindrance. All these structural and functional findings are in accordance with the notion that “trisaccharide containing α2,3-linked sialic acid” serves as a necessary and sufficient receptor unit for MuV.

## 4. Glycan Receptors with the Trisaccharide Core for MuV

The fucosylated trisaccharide (Neu5Acα2,3Galβ1,4(Fucα1,3)GlcNAc) at GlcNAc-3 connected by α1,3-linkage and the GalNAc-modified trisaccharide (Neu5Acα2,3(GalNAcβ1,4)Galβ1,4GlcNAc) at Gal-2 connected by β1,4-linkage are motifs of MuV glycan receptors (Figure 2A, motif 2 and 3) [48]. The motifs are commonly known as sialyl Lewis x (sLe^x^) and Sd^a^-glycan, respectively. The non-reducing terminal motif of Sd^a^-glycan, in which GalNAc and Neu5Ac are both linked to Gal, is known as the oligosaccharide epitope of GM2 ganglioside (Neu5Acα2,3(GalNAcβ1,4)Galβ1,4GlcCer) [12]. Structural analysis of MuV-HN in complex with sLe^x^ pentaose (Neu5Acα2,3Galβ1,4(Fucα1,3)GlcNAcβ1,3Gal) showed that the branched fucose (Fuc-3′) interacts with Tyr369 of MuV-HN (Figure 2A, motif 2). Furthermore, the complex structure of MuV-HN bound to GM2-ganglioside sugar (GM2-GS: Nue5Acα2,3(GalNAcβ1,4)Galβ1-4Glc) confirmed the interaction between the branched GalNAc moiety (GalNAc-2′) and the side chains of Glu569 and Ser539 (Figure 2A, motif 3) [48]. The interactions observed in the MuV-HN−3′-SL complex were found to be completely conserved in the trisaccharide moieties (Neu5Acα2,3Galβ1,4GlcNAc or Neu5Acα2,3Galβ1,4Glc) of sLe^x^ pentaose and GM2-GS (Figure 2A). This conservation indicated that while Neu5Acα2,3Galβ1,4Glc (or GlcNAc) constitutes the core receptor for MuV, the receptor-binding pocket, surrounded by a six-bladed-propeller fold of the MuV-HN head domain, allows for flexibility to accommodate spatially modified glycans onto the core trisaccharide receptor motif. Pre-incubation of MuV virions with 3′-SL, sLe^x^ pentaose, or GM2-GS inhibited viral entry into the cultured cell line, with the greatest inhibitory effect in the case of sLe^x^ pentaose, followed by those of 3′-SL and GM2-GS [48]. The amino acid residues involved in the interaction with glycan receptors were conserved in the MuV-HN of all genotypes except Phe370, which forms a stacking interaction with Tyr369 and is replaced with leucine in genotype K, suggesting that MuVs of all 12 genotypes utilize them as receptors.

## 5. Distribution of Glycan Receptors for MuV in the Human Body

The glycan structures involved in systemic infection by MuV may include 3′-sialyllactosamine (3′-SLN: Neu5Acα2,3Galβ1,4GlcNAc), 3′-SL, and sLe^x^ [36,48]. 3′-SLN (Figure 2C, motif 1) is present as a non-reducing terminal glycan structure of *O*- and *N*-linked glycoproteins and constitutes the core trisaccharide moiety of sLe^x^ pentaose. It exhibits a glycan structure in which glucose at the third position from the non-reducing terminal sialic acid of 3′-SL (Figure 2C, motif 1) is acetylated; despite the structural difference in the third sugar from their non-reducing termini, both 3′-SLN and 3′-SL function as core-receptor units for MuV [36]. 3′-SL constitutes the non-reducing terminal structure of a ganglioside called GM3, which is the main ganglioside present in the majority of vertebrate extraneuronal tissues, including the liver, kidney, muscles, adipose tissues, blood cells, and thyroid gland [53]. The affinity of MuV-HN to 3′-SLN suggests that MuV utilizes the sialyl-trisaccharide motif at the non-reducing terminus of both *O*- and *N*-linked glycans present in various tissues in the human body. Alternatively, some viruses, such as adeno-associated virus serotype 4 (AAV4) and serotype 5 (AAV5) show specific affinity to *O*- or *N*-linked glycans; AAV4 binds to *O*-linked glycans terminated with α2,3-linked sialic acid, whereas AAV5 binds to *N*-linked glycans terminated with α2,3- or α2,6-linked sialic acid [19,20].

sLe^x^ (Figure 2C, motif 2) is typically found on the surface of lymphocytes and neutrophils, where it plays a role in cell attachment and infiltration via recognition by selectins expressed on vascular endothelia [54,55]. Although the expression of sLe^x^ in normal human tissues is supposed to be very low, some clues indicate its expression in oral mucosa and breast tissues [56] and in heart, kidney, and lung tissues (http://www.functionalglycomics.org, 2 August 2021). sLe^x^ is also found in highly glycosylated mucins and sphingolipids in their non-reducing terminal structures [57]. It had initially been recognized as a tumor-associated antigen by the monoclonal antibody CSLEX1, which reacts specifically with various tumor tissues [58]. High expression levels of sLe^x^ antigen have been shown to correlate with invasion and metastasis of tumor cells, leading to poor prognosis of the disease [59]. Since systemic infection by MuV occurs following the infection of regional lymph nodes adjacent to the respiratory tract, and since immune cells carry glycans with sLe^x^ motifs, the immune cells may function as “carriers” to spread the virus throughout the body.

GM2-GS (Figure 2C, motif 3) is likely to be involved in neural infection by MuV [48]. GM2 is a ganglioside found in the mammalian CNS, peripheral nerves, adrenal glands, and tumors, such as melanoma, glioblastoma, and renal cell carcinoma [12,60,61]. GM1, GD1a, GD1b, GT1b, and GQ1b constitute 95% of gangliosides in the normal human brain, whereas GM2 is found in the remaining 5% of gangliosides [62,63]. Since GM2-GS is relatively less effective than sLe^x^ and 3′-SL in inhibiting MuV entry [48], the receptor function of GM2 may be limited to an environment with a high viral titer or the receptor efficacy of GM2 may be relatively low.

Recent studies have revealed the details of glycan structures that MuV prefers for its receptors. However, the distribution of glycan receptors in the human body alone cannot explain all the unique tropism exhibited by MuV; there may be additional unknown host factors that promote MuV infection.

## 6. Envelope Glycoproteins and Entry Models of Paramyxoviruses

**Figure 3:**
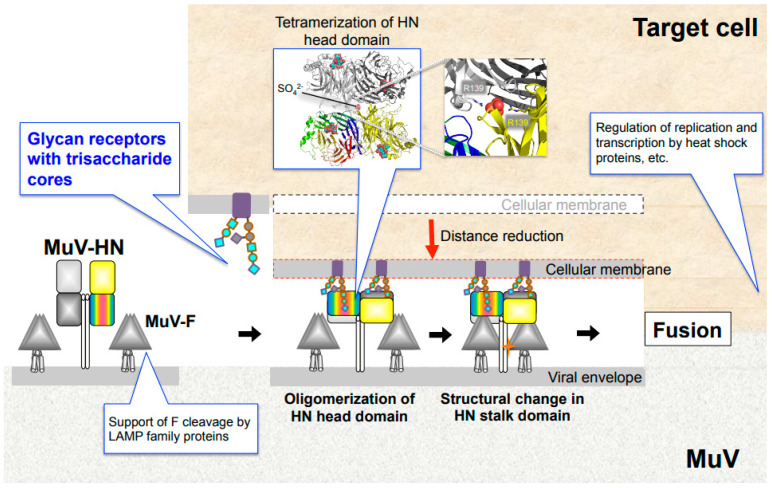
MuV entry mechanism and host factors. Binding of MuV-HN to glycan receptors causes sequential conformational changes in the HN and F proteins, leading to membrane fusion. Multimerization of the MuV-HN head domain upon receptor binding may affect the structural change of the MuV-HN stalk domain, which in turn may trigger the structural change of the F protein. Following cellular entry, replication and transcription of the viral genome are regulated by host factors, such as heat shock protein family. LAMP family proteins support furin-mediated cleavage of the newly synthesized F protein.

The attachment proteins of paramyxoviruses consist of an N-terminal cytoplasmic tail, a transmembrane region, a stalk region, and a C-terminal head domain. Two attachment protein dimers form a tetramer, which is generally called a “dimer-of-dimers. Paramyxovirus entry is initiated by the attachment of HN/H/G proteins to their cellular receptors. Upon receptor binding, the paramyxovirus attachment protein undergoes structural/conformational changes that sequentially stimulate structural changes in the adjacent F protein [10]. This structural change of the F protein provides the force that leads to the fusion of the viral envelope with the cellular membrane, allowing the viral genome to enter the host cell (Figure 3). Several models have indicated that F protein activation is triggered by the receptor binding of attachment proteins [10,64,65,66,67,68]. Crystal structures of the HN-protein ectodomains from NDV and PIV5 suggest that receptor binding induces a conformational change in the attachment protein from a “four-head down” to “four-head up” position, resulting in the exposure of the stalk domain that can then interact with the F protein [69,70]. Studies have suggested that part of the exposed stalk domain of the HN protein is critical for activation of the F protein. Importance of the stalk region in triggering fusion is likely to be common among paramyxoviruses, including viruses that use glycan receptors (PIVs, NDV, MuV, etc.) and proteinaceous receptors (morbiriviruses and henipaviruses) for their attachment [71,72,73,74,75,76,77]. Conformational changes in the dimer and/or tetramer of the attachment protein also regulate fusion-triggering processes. Studies on hPIV3 and NDV, which utilized biomolecular fluorescence complementation techniques and X-ray crystallography, have shown that the HN dimer interfaces are critical for HN-F interaction and F activation [78,79,80,81]. Structural and functional studies on MeV and MuV have shown conformational changes in the head domain that trigger the activation of F proteins. In MeV, receptor binding has been shown to trigger rearrangement of the H protein between two forms of tetrameric assembly of the head domain (form I and form II) [77]. Mutations in MeV-H at the dimer–dimer interfaces of form I and form II inhibit cell fusion induced by the function of the MeV-F protein [82]. The MuV-HN head domain forms a tetramer composed of the dimer-of-dimers similar to other paramyxovirus attachment proteins using sialic acid-containing glycan as a receptor. The dimer-of-dimers conformation of the MuV-HN head domain is characterized by the presence of a divalent anion at the center of two dimer interfaces (Figure 3) [83]. The anion was found to act as a “linker” between the two dimers, interacting with the side chain of positively charged arginine residues at position 139 (Arg139) of the MuV-HN protein. Mutations in Arg139 of the MuV-HN protein decreased the ability of the F protein to induce fusion, suggesting tetramerization of the MuV-HN head domain to be crucial for triggering fusion. Altogether, multimerization of the head domain of the attachment protein is critical for triggering fusion in several paramyxoviruses that use glycan receptors or proteinaceous receptors. In other words, although the molecular mechanism of multimerization may differ across viruses, suitable multimerization of the head domain of attachment proteins may play an essential role in triggering fusion in paramyxoviruses.

Two major differences in the cell entry of paramyxoviruses include the following. First, the H protein of morbiliviruses and the G protein of henipaviruses bind to proteinaceous receptors, whereas the HN protein binds to sialic acid-containing glycan receptors [10]. Second, binding of proteinaceous receptors by H/G proteins is thought to release F proteins from preassembled H-F oligomers, allowing for spontaneous structural changes in the F proteins [64,84,85,86]. In contrast, binding of HN proteins to sialic acid-containing glycan receptors is believed to induce structural changes by actively interacting with F proteins [64,84,86,87]. Taken together, although there are some common mechanisms of cell entry among paramyxoviruses, the mechanisms leading to the triggering of fusion may differ at some point from those of paramyxoviruses that utilize either proteinaceous receptors or sialic acid-containing glycan receptors.

## 7. Host Factors for MuV

### 7.1. Lysosome-Associated Membrane Proteins

The lysosome-associated membrane protein (LAMP) family may be a host factor that contributes to MuV tropism. A recent study revealed that LAMPs indirectly support the cleavage of the MuV-F protein to convert the inactive F_0_ precursor into the active F_1_-F_2_ heterodimer [88]. Cleavage of F_0_ into F_1_-F_2_ is indispensable for the membrane fusions of virus-to-cell or infected cell-to-neighboring cells in paramyxoviruses, such as MuV, MeV, hPIV3, PIV5, and NDV virulent strains. The F proteins of these viruses are specifically recognized by the cellular protease furin and cleaved at the R-X-K/R-R consensus sequence [10,89,90,91,92,93]. Despite the fact that 293T cells express not only glycan receptors of MuV but also furin, little cell-to-cell fusion was found to occur in 293T cells upon MuV infection or by co-expression of MuV-HN and F proteins, due to the insufficient cleavage of F_0_ into the F_1_-F_2_ form. Interestingly, overexpression of LAMPs (LAMP1, LAMP2A, LAMP2B, or LAMP3) complements the failure of F processing and confers the 293T cells with the ability to produce syncytia [88]. The LAMP family, which comprises LAMP1, LAMP2, LAMP3 (also called CD208 or dendritic cell (DC)-LAMP), LAMP4 (also called CD68 or macrosialin), and LAMP5 (also called brain and dendritic cell (BAD)-LAMP), are type-1 transmembrane proteins that localize predominantly in lysosomes [94,95,96]. The LAMP family members are involved in multiple cellular functions, including the regulation of lysosomal cell death, autophagy, and phagocytosis [97]. LAMP2 undergoes alternative splicing to produce the isoforms LAMP2A, B, and C [98]. LAMP1 and LAMP2 are the most abundant proteins in the lysosomal membrane, whereas LAMP3 is highly specific in mature DCs in humans. LAMP3 is located in the MHC class II compartment and has been proposed to regulate lysosomal function after the peptide-MHC class II molecules are transferred to the cell surface [99]. In addition, LAMP3 is induced by interferon in various cells [100]. LAMP3 may be the most critical for the MuV-F cleavage process, among the LAMP members, because it is the most efficient at supporting MuV-F cleavage compared to the others. Endogenous LAMP3 gene expression in 293T cells is much lower than that in parental HEK293 cells, in which cell-cell fusion occurs efficiently by co-expression of MuV-HN and F proteins. LAMPs interact with both furin and the MuV F_0_ precursor, which suggests that they may convert F_0_ into a suitable substrate structure for furin or serve as a scaffold for MuV-F and furin [88]. Future studies can clarify whether LAMP-mediated processing of the F protein by furin can be a common mechanism for other viruses, or whether it is a MuV-specific phenomenon.

### 7.2. Heat Shock Proteins

Heat shock proteins (Hsps) are the ubiquitously expressed chaperones that play a role in protein folding, regulation of protein turnover, apoptosis, and autophagy. They also perform protective functions against infections by stimulating innate and adaptive immunity [101]. In viral infections, some Hsps have been reported to exhibit pro- and anti-viral functions at various steps of viral life cycles including viral entry, disassembly, replication, and assembly; for example, the stress-induced 70 kDa heat shock protein (Hsp70) assists replication or translation of the viral genomes of human hepatitis B virus, human immunodeficiency virus, West Nile virus, dengue virus, Ebola virus, and enterovirus A71 [102,103]. Hsp90 is also required for viral replication of many different viruses in cooperation with Hsp70 and/or other additional factors to regulate the process [104]. Recent studies have revealed that Hsp70 and Hsp90 play a role in regulating MuV replication and transcription. Among the human Hsp70 family, which comprises at least eight unique gene products [105], Hsp72 is mobilized to the cytoplasmic inclusion bodies of infected cells where the MuV-P protein, an essential protein for viral RNA synthesis [10], is recruited by Hsp72 and degraded through the ubiquitin–proteasome pathway [106]. The regulatory effects of Hsp70 family proteins on viral propagation have also been reported in MeV; Hsp72 increases the transcription and replication of MeV through its interaction with N protein of the Edmonston strain [107]. The MuV-L protein, together with the MuV-P protein, forms part of the viral RNA-dependent RNA polymerase. Based on the observation that Hsp90 associated with the MuV-L protein but not with the MuV-P protein, and that the MuV-L protein is degraded in the presence of Hsp90 inhibitors in MuV-infected cells, Hsp90 is believed to play an important role in the stability and function of the MuV-L protein prior to formation of the mature polymerase complex with the MuV-P protein. Degradation of the MuV-L protein is carried out through the carboxyl terminus of the Hsp70-interacting protein (CHIP)-mediated proteasomal pathway [108]. A recent study also revealed that the R2TP complex, a co-chaperone of Hsp90 [109], is another host factor that regulates the transcription of MuV and MeV by interacting with each L protein. It was shown that the R2TP complex suppresses RNA synthesis of these two viruses. For MeV, it negatively affects propagation. In contrast, for MuV, the regulation by the R2TP complex allows the virus to evade innate immune responses and ensures the MuV replication [110].

## 8. Future Investigations

MuV utilizes α2,3-sialyl-trisaccharide as its core-receptor structure to enter target cells (Figure 2A,C, motif 1). It can flexibly bind to glycans with modifications of the core structure, such as sLe^x^ and GM2 (Figure 2A,C, motif 2 and 3) [48]. Following cellular entry, MuV replication and transcription are optimized by interaction with host factors, such as Hsp70 and Hsp90 (Figure 3) [106,108]. For newly expressed glycoproteins in the infected cell, LAMPs cooperate with the protease furin to support the cleavage of MuV-F protein from F_0_ to form F_1_-F_2_ (Figure 3) [88]. Although the distribution of these receptors and host factors may partially explain MuV tropism, many questions still need to be addressed to fully understand the pathogenesis of MuV: Why is the affinity to glandular tissues and the CNS unique to MuV, despite its similar life cycle to other paramyxoviruses? Are the tissues damaged by direct infection of the organs by MuV or by the secondary effect of immune response? Are there other receptors or host factors that restrict the MuV life cycle in specific tissues? These are just some of the questions that need further attention. In future, addressing these questions will facilitate better understanding of the unique tropism and pathogenesis of MuV and the development of effective therapeutic strategies.

## Data Availability

Not applicable.
